# Particulate Matter from Both Heavy Fuel Oil and Diesel Fuel Shipping Emissions Show Strong Biological Effects on Human Lung Cells at Realistic and Comparable *In Vitro* Exposure Conditions

**DOI:** 10.1371/journal.pone.0126536

**Published:** 2015-06-03

**Authors:** Sebastian Oeder, Tamara Kanashova, Olli Sippula, Sean C. Sapcariu, Thorsten Streibel, Jose Manuel Arteaga-Salas, Johannes Passig, Marco Dilger, Hanns-Rudolf Paur, Christoph Schlager, Sonja Mülhopt, Silvia Diabaté, Carsten Weiss, Benjamin Stengel, Rom Rabe, Horst Harndorf, Tiina Torvela, Jorma K. Jokiniemi, Maija-Riitta Hirvonen, Carsten Schmidt-Weber, Claudia Traidl-Hoffmann, Kelly A. BéruBé, Anna J. Wlodarczyk, Zoë Prytherch, Bernhard Michalke, Tobias Krebs, André S. H. Prévôt, Michael Kelbg, Josef Tiggesbäumker, Erwin Karg, Gert Jakobi, Sorana Scholtes, Jürgen Schnelle-Kreis, Jutta Lintelmann, Georg Matuschek, Martin Sklorz, Sophie Klingbeil, Jürgen Orasche, Patrick Richthammer, Laarnie Müller, Michael Elsasser, Ahmed Reda, Thomas Gröger, Benedikt Weggler, Theo Schwemer, Hendryk Czech, Christopher P. Rüger, Gülcin Abbaszade, Christian Radischat, Karsten Hiller, Jeroen T. M. Buters, Gunnar Dittmar, Ralf Zimmermann

**Affiliations:** 1 HICE—Helmholtz Virtual Institute of Complex Molecular Systems in Environmental Health—Aerosols and Health, www.hice-vi.eu, Neuherberg, Rostock, Munich, Karlsruhe, Berlin, Waldkirch, Germany, Kuopio, Finland, Cardiff, UK, Esch-Belval, Luxembourg; 2 Center of Allergy and Environment (ZAUM), Helmholtz Zentrum München and Technische Universität München, Member of the German Center for Lung Research (DZL), Munich, Germany; 3 CK-CARE, Christine Kühne Center for Allergy Research and Education, Davos, Switzerland; 4 Mass Spectrometry Core Unit, Max Delbrück Center for Molecular Medicine Berlin-Buch, Germany; 5 University of Eastern Finland, Department of Environmental Science, P.O. Box 1627, FI-70211 Kuopio, Finland; 6 Luxembourg Centre for Systems Biomedicine, University of Luxembourg, L-4362 Esch-Belval, Luxembourg; 7 Joint Mass Spectrometry Centre, Chair of Analytical Chemistry, Institute of Chemistry, University Rostock, Rostock, Germany; 8 Joint Mass Spectrometry Centre, CMA—Comprehensive Molecular Analytics, Helmholtz Zentrum München, Neuherberg, Germany; 9 Institute for Technical Chemistry (ITC), Karlsruhe Institute of Technology, Campus North, Karlsruhe, Germany; 10 Institute of Toxicology and Genetics (ITG), Karlsruhe Institute of Technology, Campus North, Karlsruhe, Germany; 11 Chair of Piston Machines and Internal Combustion Engines, University Rostock, Rostock, Germany; 12 VTT Technical Research Centre of Finland, P.O. Box 1000, FI-02044 VTT, Espoo, Finland; 13 National Institute for Health and Welfare, Department of Environmental Health, P.O. Box 95, FI-70701, Kuopio, Finland; 14 Institute of environmental medicine, UNIKA-T, Technische Universität, Munich, Germany; 15 Lung and Particle Research Group, School of Biosciences, Cardiff University, Cardiff, Wales, United Kingdom; 16 Research Unit Analytical BioGeoChemistry, Helmholtz Zentrum München—German Research Center for Environmental Health GmbH, Neuherberg, Germany; 17 Vitrocell GmbH, Waldkirch, Germany; 18 Laboratory of Atmospheric Chemistry, Paul Scherrer Institute (PSI), Villigen, Switzerland; 19 Institute of Physics, University Rostock, Rostock, Germany; University of Alabama at Birmingham, UNITED STATES

## Abstract

**Background:**

Ship engine emissions are important with regard to lung and cardiovascular diseases especially in coastal regions worldwide. Known cellular responses to combustion particles include oxidative stress and inflammatory signalling.

**Objectives:**

To provide a molecular link between the chemical and physical characteristics of ship emission particles and the cellular responses they elicit and to identify potentially harmful fractions in shipping emission aerosols.

**Methods:**

Through an air-liquid interface exposure system, we exposed human lung cells under realistic *in vitro* conditions to exhaust fumes from a ship engine running on either common heavy fuel oil (HFO) or cleaner-burning diesel fuel (DF). Advanced chemical analyses of the exhaust aerosols were combined with transcriptional, proteomic and metabolomic profiling including isotope labelling methods to characterise the lung cell responses.

**Results:**

The HFO emissions contained high concentrations of toxic compounds such as metals and polycyclic aromatic hydrocarbon, and were higher in particle mass. These compounds were lower in DF emissions, which in turn had higher concentrations of elemental carbon (“soot”). Common cellular reactions included cellular stress responses and endocytosis. Reactions to HFO emissions were dominated by oxidative stress and inflammatory responses, whereas DF emissions induced generally a broader biological response than HFO emissions and affected essential cellular pathways such as energy metabolism, protein synthesis, and chromatin modification.

**Conclusions:**

Despite a lower content of known toxic compounds, combustion particles from the clean shipping fuel DF influenced several essential pathways of lung cell metabolism more strongly than particles from the unrefined fuel HFO. This might be attributable to a higher soot content in DF. Thus the role of diesel soot, which is a known carcinogen in acute air pollution-induced health effects should be further investigated. For the use of HFO and DF we recommend a reduction of carbonaceous soot in the ship emissions by implementation of filtration devices.

## Introduction

Epidemiological studies provide compelling evidence that pollution by airborne particulate matter (PM) derived from fossil fuel combustion is an important cause of morbidity and premature death [1, 2]. Chronic PM exposure can induce short-term (e.g., cardiovascular diseases or asthma) and long-term health effects, most notably cancer. Diesel automobile emissions were recently classified as human carcinogens by the International Agency for Research on Cancer [3].

Diesel ship emissions substantially contribute to worldwide anthropogenic PM levels, which account for up to 50% of the PM-related air pollution in certain coastal areas, rivers and ports [4–7]. Epidemiological studies attribute up to 60,000 annual deaths from lung and cardiovascular disease [8] to ship engine PM. A variety of new regulations will soon be implemented to ensure cleaner ship emissions [9–11]. Low-grade heavy fuel oils (HFOs) contain high levels of sulphur, toxic polycyclic aromatic hydrocarbons (PAHs) and transition metals. Current regulations target HFO use by limiting their sulphur content. In this context, the maximum sulphur content in shipping fuel is internationally regulated by the International Maritime Organisation (IMO) at 3.5%; in most European and US coastal areas, the maximum allowed sulphur content is 1% (Sulphur Emission Control Areas, SECA) [12, 13]. Furthermore, in 2015, a 0.1% sulphur fuel limit will be implemented in the Baltic and North Sea SECAs [14]. To comply with these new sulphur limits, highly refined distillate fuels are necessary (diesel fuel, DF, or marine gas oil, MGO). Currently, MGO is the most used distillate fuel for marine shipping and contains up to 1% sulphur. In 2011, 170 million tons of HFO and 43 million tons of MGO and DF were consumed by ship diesel engines worldwide [15, 16]. This volume corresponds to approximately 21% of global fuel consumption [17].

The biological and health effects of land-based diesel engine emissions have been extensively studied using submersed cell cultures subjected to collected diesel exhaust particles [18, 19]. This submersed cell culture approach neglects the effect of airborne particle exposure, which can result in low sensitivity in measuring biological effects [20]. An alternative is the air-liquid interface (ALI) cell exposure technology. Current systems are technically mature enough to enable reproducible, direct, on-site exposure of lung cell culture to emission aerosols under realistic dilution, flow and humidity conditions [21]. Multiple ALI-exposure studies using car diesel engines [22–25] highlight the improved sensitivity of ALI systems compared with submerged toxicological test systems that use collected diesel exhaust particles (DEP) [20].

Up to now three main causes for PM-induced health effects have been identified: genotoxicity, inflammation and oxidative stress; other mechanisms have also been described [19]. Thus far, all information on diesel PM has been inferred from research on car emissions. However, diesel emissions from ships differ greatly from car or truck diesel emissions due to the fuel composition (HFO) and combustion characteristics of ship engines [26]. Thus, the practice currently used to estimate the health impacts of ship diesel emissions based on analogous car or truck emissions [8, 12, 27] is problematic. The high levels of toxic compounds [6, 28] suggest that HFO emissions produce more detrimental acute and chronic toxic effects than car or truck diesel emissions.

This study targets the biological effects of airborne PM from both diesel and HFO ship emissions based on their chemical compositions. The joint analysis of the biological multi-omics data with the comprehensive aerosol analysis results provides an extensive overview of affected biological mechanisms and pathways and further identifies potentially harmful fractions of the shipping aerosols.

## Results and Discussion

### Experimental setup

**Fig 1:**
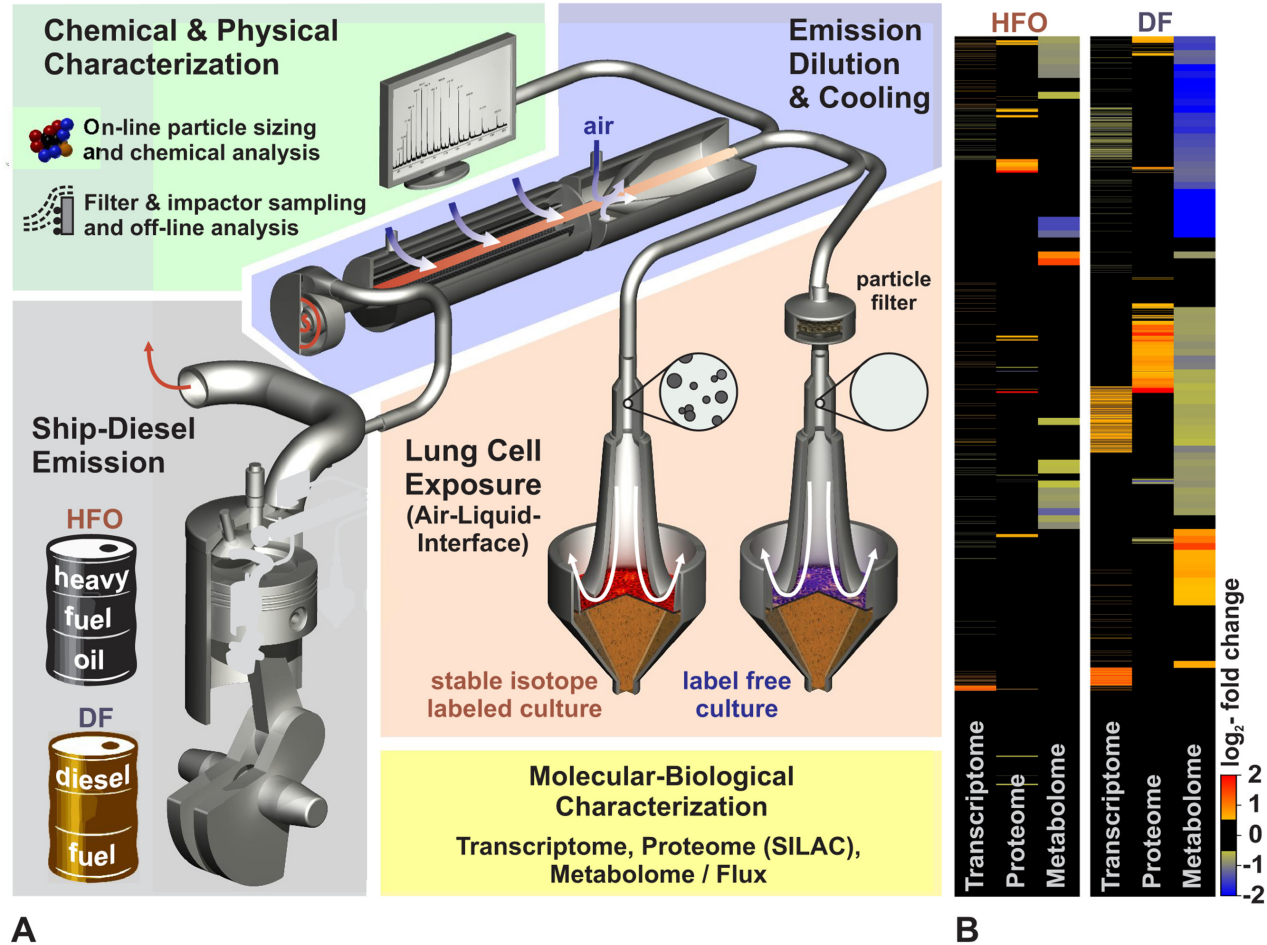
Experimental set-up and global omics analyses. (A) An 80 KW common-rail-ship diesel engine was operated with heavy fuel oil (HFO) or refined diesel fuel (DF). The exhaust aerosols were diluted and cooled with clean air. On-line real-time mass spectrometry, particle-sizing, sensor IR-spectrometry and other techniques were used to characterise the chemical composition and physical properties of the particles and gas phase. Filter sampling of the particulate matter (PM) was performed to further characterise the PM composition. Lung cells were synchronously exposed at the air-liquid-interface (ALI) to aerosol or particle-filtered aerosol as a reference. The cellular responses were characterised in triplicate at the transcriptome (BEAS-2B), proteome and metabolome (A549) levels with stable isotope labelling (SILAC and ^13^C_6_-glucose). (B) Heatmap showing the global regulation of the transcriptome, proteome and metabolome.

**Fig 2:**
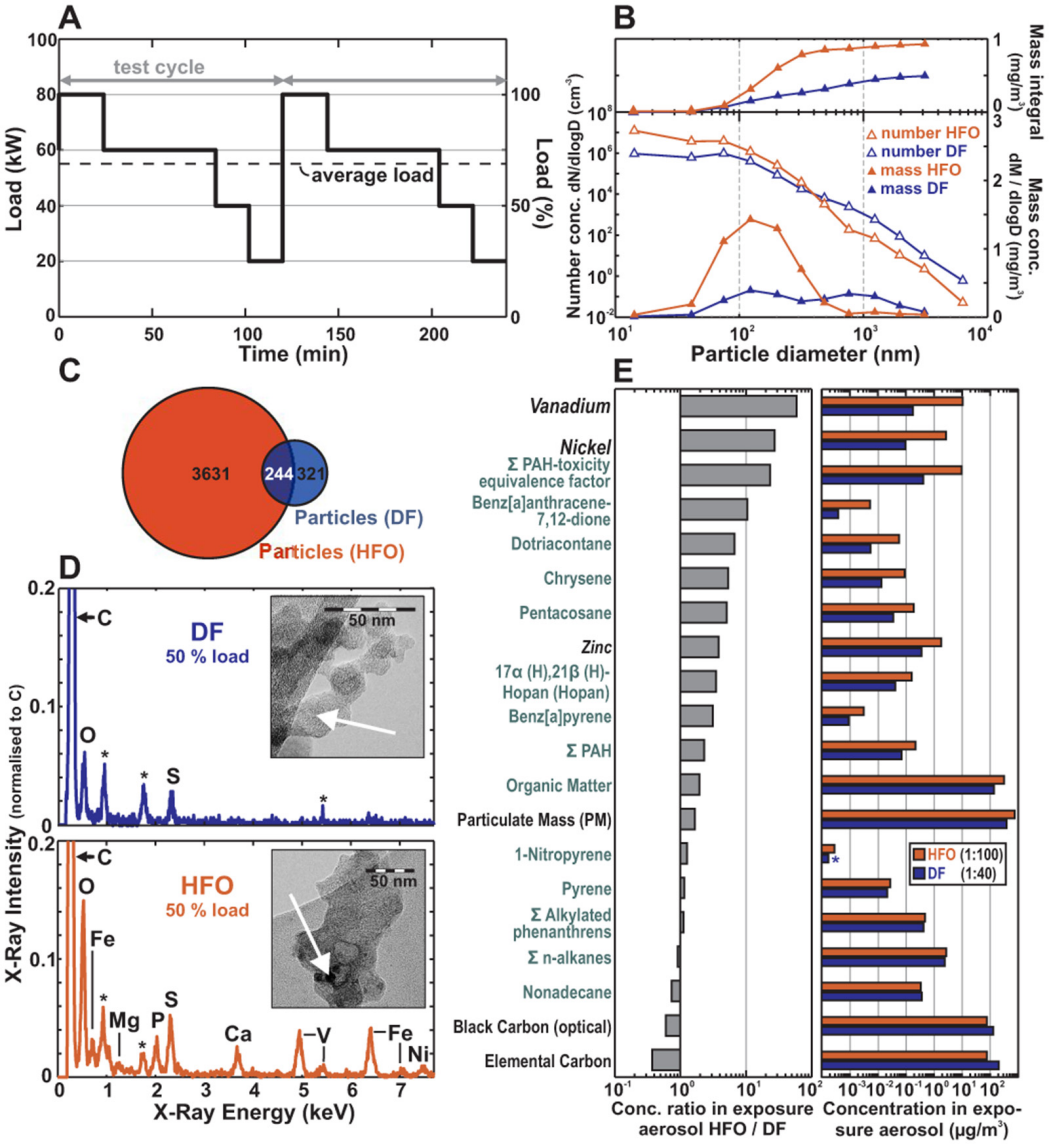
Chemical and physical aerosol characterisation. (A) The ship diesel engine was operated for 4 h in accordance with the IMO-test cycle. (B) Approximately 28 ng/cm^2^ and 56 ng/cm^2^ were delivered to the cells from DF and HFO, respectively, with different size distributions. The HFO predominantly contained particles <50 nm, and the DF predominantly contained particles >200 nm, both in mass and number. (C) Number of chemical species in the EA particles. (D) Transmission electron microscope (TEM) images and energy-dispersive X-ray (EDX) spectra of DF-EA and HFO-EA; heavy elements (black speckles, arrow); and contributions of the elements V, P, Fe and Ni in the HFO particles using EDX (* = grid-material). (E) Exemplary EA concentrations (right) and concentration ratios (left) for particulate matter-bound species. For all experiments, n = 3.

The experimental setup is illustrated in Fig 1 (details in S1 and S2 Figs and in S1 Text). Briefly, we operated a four-stroke, one-cylinder common rail research ship diesel engine (80 kW) using either HFO (HFO 180) containing 1.6% sulphur or DF containing less than 0.001% sulphur and 3.2% plant oil methyl ester in compliance with the 2014 IMO-SECA-legislation (DIN EN590, see S1 Fig for the engine and fuel properties), which represents the common dual-fuel use in commercial shipping [10, 29]. The engine was operated according to the test cycle ISO 8178–4 E2 for ship diesel engines with a balance between harbour-manoeuvring and cruising engine-loads (Fig 2). The combustion aerosol was cooled and diluted with sterile air. Chemical and physical properties of the HFO and DF aerosol were comprehensively characterised using state-of-the-art, on-line and real-time techniques as well as off-line filter sample analyses. Results are summarised in Fig 2 (for details, see SI). In parallel with aerosol characterisation, confluent layers of two human epithelial lung cell lines (the human lung alveolar cancer cell line A549, purchased from the American Type Culture Collection, ATCC CCL-185; http://www.lgcstandards-atcc.org/Products/All/CCL-185.aspx, and human SV40-immortalised bronchial epithelial cells BEAS-2B, purchased from ATCC, CRL-9609; http://www.lgcstandards-atcc.org/Products/All/CRL-9609.aspx)) [30] were exposed to the diluted engine exhaust for 4 h at the ALI (Fig 1). Epithelial lung cells have direct contact to inhaled aerosol particles and gases and were therefore used as a model of aerosol inhalation. The cell lines A549 and BEAS-2B have been widely used for testing particles and gases at the air-liquid-interface [31–36]. The BEAS-2B cells are considered to better resemble the situation in human lung tissue while requirements for the cultivation of the cancer derived cell line A549 are better suited for labeling with the L-D_4_-Lysine isotope maker for the quantitative proteomics. The transcriptomics methodology is not based on metabolic labelling and thus well suited for the analysis of BEAS-2B cells. The quantitative comparative proteomics approach requires the labelling of the cells with D4-Lysine. However the BEAS-2B cells require specialized media and coating of the plates, which is currently incompatible with the metabolic labelling. Therefore simultaneuos SILAC-based proteomic and metabolic analysis was performed with the established A549 cell model. In summary the cells were analysed using transcriptome (BEAS-B), SILAC-proteome (A549), metabolome and metabolic flux measurements (A549) as well as cytotoxicity tests (A549). The omics data are stored in Gene Expression Omnibus (GSE63962) and Proteomics DB (PRDB004215), respectively. All experiments were performed in triplicate (3 independent exposures) and referenced to filtered aerosol (for normalising the effects induced by the gas phase) because particles and particle-related chemicals play an important role in the health relevance of diesel exhaust [37] and are therefor in the focus of this study.

The first phase of the experiment was used to find the optimal dose for the large-scale analysis. Cells in the setup were exposed to different concentrations of aerosols. The reaction of the cells was monitored using the Alamar Blue viability test. Due to the higher particle concentration in HFO-exhaust (see below) a dilution of 1:100 was required to achieve a non-impaired cell status while for DF-exhaust a lower dilution of 1:40 was possible without any viability impairment (i.e., a no acute toxicity exhaust dilution; S3A Fig). By applying the different dilution ratios of 1:40 (DF) and 1:100 (HFO) for the exhaust gases for no acute toxicity at 4 h exposure, a similar deposition dose (deposited particle mass per confluent cell culture surface area, see below) was achieved. Based on a gravimetric filter analysis of PM 2.5 and assuming a size-independent, constant deposition probability of 1,5% after Comouth et al. [38], the accumulated particle mass deposited on the lung cell monolayer surface area was roughly estimated as 28 ± 1.5 (DF) and 56 ± 0.7 ng/cm^2^ (HFO) per 4 h exposure duration (see S3C Fig) with the variance of the mass measurement expressed by the standard deviation of the filter samples. A more elaborated model taking into account the particle size distribution from an electric low pressure impactor (ELPI) and a size dependent deposition probability after Comouth et al. [38], which was determined using previous measurements from ALI exposure systems, predicts 15.7 (DF) and 41.5 ng/cm^2^ (HFO) per 4 h exposure. Even for improved deposition approximation model, the estimated uncertainties, however, are rather high (about a factor of 2). Therefore the deposition dose in both cases can be considered being approximately equal for DF and HFO. We decided to perform the exposure for omics measurements at these dilutions, in order to compare the specific molecular biological effect strength at an about equal deposition dose. Note that in the following all aerosol parameters are reported considering the specific emission-aerosol dilution factors (i.e. the exposure aerosol, EA, as delivered to the cells).

### Chemical and physical analysis

Consistent with previous studies [29], only small concentration differences of gaseous compounds were found in the emissions of the ship engine using the two fuels. An exception was SO_2_ (4 mg/m^3^), which was below toxicity threshold after dilution in the HFO-EA. In addition, the EA concentrations of the further potentially toxic gases NO, NO_2_ and CO were below 16.3, 0.4 and 7 ppm, respectively. These values are below the reported toxicity thresholds for the air-liquid interface [39, 40] and even below the general NIOHS lifetime workplace 8-hr exposure limit values of 25, 1 and 35 ppm, respectively [41].

The concentration of particles with an aerodynamic diameter larger than 200 nm was higher for the DF-EA (particle number and mass concentration), whereas nanoparticles smaller than 50 nm were approximately 100-fold more abundant for the HFO-EA (see the size distributions in Fig 2). However, note that the mass contribution of these nanoparticles is very small. TEM images of the particles show that the smaller HFO particles (Fig 2) contained high levels of amorphous organic material around carbonaceous fractal cores with metal inclusions. The DF-EA particle analysis reveals a different picture (Fig 2), in which the particles appear larger and are mostly composed of pure carbonaceous aggregates with spherical soot cores (Ø ~ 20–30 nm). A layered graphite-like carbon structure became visible at a higher TEM magnification (S3 Fig). Based on the size-dependent deposition function described by Comouth *et al*. [38] (S3 Fig) and the low specific density of the observed fractal soot aggregates in DF-EA (Fig 2), the deposited mass for the DF-EA cell exposure experiments is slightly lower than the above estimates. The particles deposited from the HFO-EA were of a higher dose in mass and number compared to the DF-EA exposure.

Energy-dispersive X-ray spectroscopy (EDX, Fig 2) on TEM showed large differences between HFO-EA and DF-EA particles with regard to the abundance of heavy elements. High intensities of elements such as vanadium, nickel, sulphur and iron were detected in the HFO particles, whereas the DF particles primarily contained carbon and oxygen in the EDX spectrum. Fig 2 shows an overview of the differences in the inorganic and organic chemical composition (Fig 2) as well as the absolute concentrations of the respective substances in the DF- and HFO-exposure aerosol particles (Fig 2 and S4 Fig). Almost all of the measured components, except elemental carbon and black carbon, were more abundant in HFO-EA compared with DF-EA, despite a 2.5-fold higher dilution for HFO-EA.

On-line aerosol mass spectrometry and off-line analyses showed considerably higher mass concentrations of particle-bound organic material and much more complex organic material in the HFO-EA (S1 Table). High-resolution mass spectrometry (ESI-FTICR-MS) revealed 3631 different polar organic compounds in the HFO particles compared with only 321 in the DF particles (Fig 2); 244 compounds were common to both fuel types. The quantification of aromatic and aliphatic compounds (S4 Fig) revealed that higher molecular weight components were more abundant in the HFO particles (green text in Fig 2), such as the higher molecular weight carcinogenic PAH benzo[a]pyrene (Fig 2 and S4 Fig). The sum of PAH toxicity equivalency factors (Fig 2), which ranks different toxic PAHs weighted by their concentration and relative toxicity, was more than 10-fold higher in HFO-PM compared with DF-PM (Fig 2). The only component over-represented in the DF-PM was the elemental carbon fraction (EC) and the corresponding optically measured “black carbon” factor (BC; Fig 2).

Summarising the chemical and physical characterisations, particles emitted from ship engines differ in concentration, size distribution, morphological appearance and chemical composition depending on whether DF or HFO is used. The DF particles in the inhalable size region were dominated by elemental carbon-rich soot-aggregate particles [29], whereas the HFO particles were smaller (nanoparticles) and rich in organic material, including known organic air toxicants (PAHs and their derivatives) and reactive transition metals such as V, Ni, Fe and Zn (S4 Fig). However, it shall be noted that also DF-PM contains organic compounds in relatively high concentrations. The HFO-PM just contains much higher concentrations (Fig 2).

### Exposure and deposition dose

We exposed human lung cells for 4 h to concentrations which are corresponding to occupational exposure scenarios or 10 times the concentration of an ambient high concentration scenario (EA ~ 390 μg DF PM2.5/m^2^ and ~760 μg HFO PM2.5/m^2^). This concentration corresponds to an ALI mass deposition dose of about 28 and 56 ng PM/cm^2^ for DF and HFO respectively.

These doses can be related to the human respiratory tract using the specific deposition efficiency for different lung regions. From the measured size distribution and an estimated effective particle density based on the mass-mobility-relation for aggregated diesel particles (between 1.1 and about 0.1 g cm^-3^, derived from [42, 43]), a deposition simulation was performed using a recently updated model [44, 45] for the tracheobronchial lung region. A 4 h exposure of a human being to the EA concentrations used in our experiments would result to a tracheobronchial deposition of about 1.5 and 5 ng PM/cm^2^ for DF and HFO, respectively. Thus the deposited mass in an ALI experiment corresponds to about 3 days (DF) or 2 days (HFO) exposure time for an exposed person (note that for an equal dilution of 1:100 in both EAs the actual deposited tracheobronchial dose for DF would correspond to a 7.5 days exposure of a person). However, one should keep in mind that the size distribution may change quickly in the ambient atmosphere and in the airways, e.g. by coagulation or water condensation, causing additional uncertainty thereby. The similarity between the size dependent deposition curve [38] for the ALI-system and for the lung deposition curve [44] suggests a good transferability of the results, in particular for the tracheobronchial region. In conclusion, the deposited mass concentration of at an equal dilution of DF PM mass would be about ¼ of the deposited HFO PM. This however, only holds true for directly emitted aerosols. In the atmosphere the more polar, sulphate containing HFO emission particle will quickly grow considerably by water condensation, while the hydrophobic DF particles size distribution will remain stable for longer time [46, 47]. Therefore, emission size distributions might, to some extent, equalise soon.

### Biological analysis

To relate the extensive chemical and physical characterisation of the exhaust aerosols to biological effects, the HFO and DF emission particles were directly deposited on human lung cells using ALI exposure technology. Transcriptome, proteome, metabolome and metabolic flux analyses were performed, which yielded parallel and relative quantification of 42205 different transcripts, 6192 proteins and 400 metabolic molecules. To reduce variability, the proteins and metabolites were extracted from the same cell material (A549) that was previously metabolically labelled using D_4_-lysine (SILAC proteomics) and ^13^C_6_-glucose (metabolic flux analysis). Ribonucleic acid (RNA) was isolated from BEAS-2B cells exposed through the same ALI exposure system and was used for the transcriptome analyses [20].

**Fig 3:**
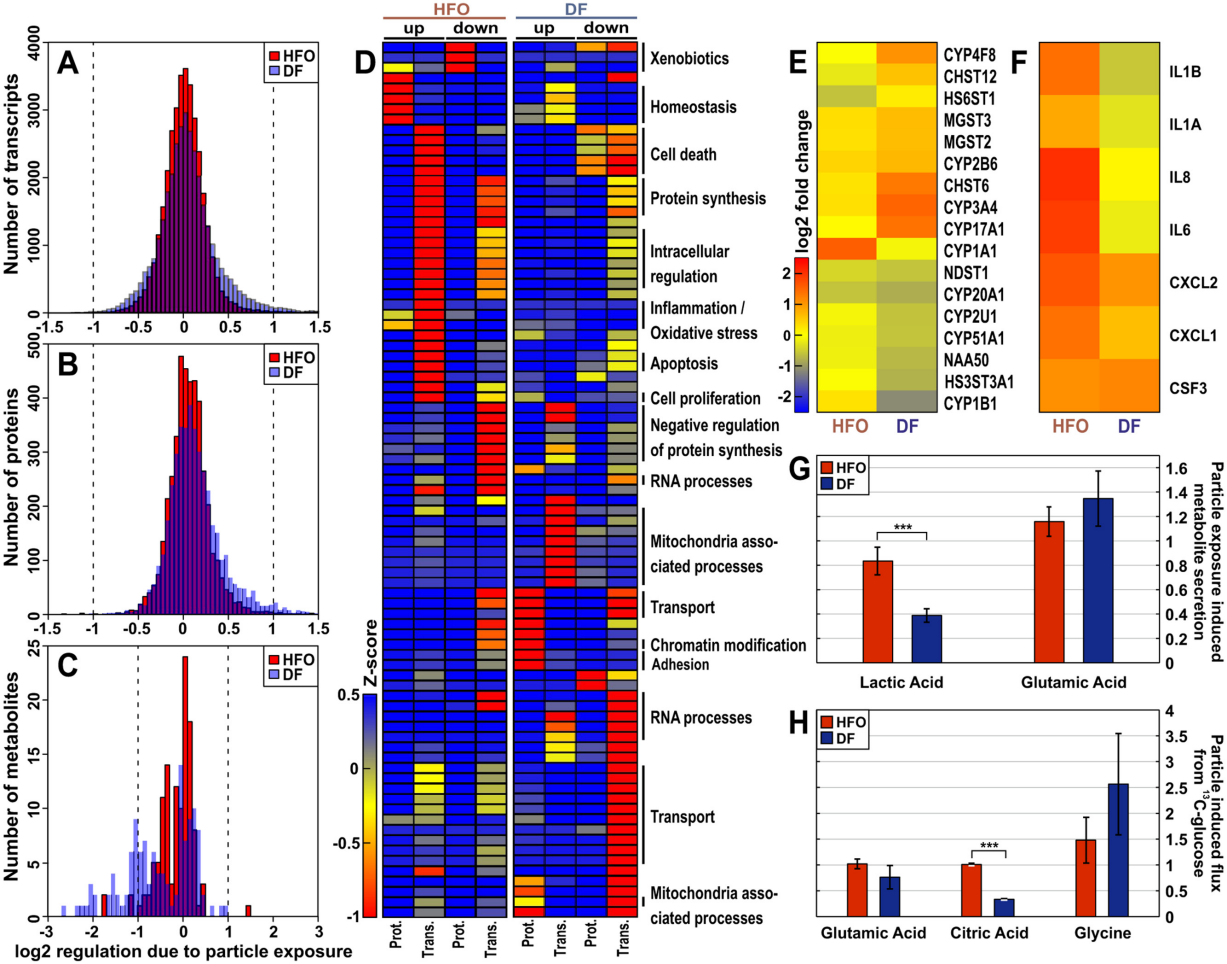
Effects of shipping particles on lung cells. The net effects from the particles were referenced against the gaseous phase of the emissions. (A) Number of the regulated components in the transcriptome shows more genes regulated by the DF than the HFO particles (in BEAS-2B cells). Similar results were observed for the proteome (B) and metabolome (C) (in A549 cells). (D) Meta-analyses for the transcriptome and proteome using the combined Gene Ontology (GO) term analysis of the 10% most regulated transcripts and proteins. Individual GO terms are listed in S2 Table; the hierarchical pathways are indicated on the right. (E) Gene regulation of Wiki-pathway bioactivation; (F) gene regulation of Wiki-pathway inflammation; g, secreted metabolites; and h, metabolic flux measurements using ^13^C-labelled glucose. For all experiments, n = 3.

The transcriptome, proteome and metabolome analyses revealed widespread changes in the cellular system upon exposure to both HFO and DF aerosol particles. Surprisingly, more gene expression levels were regulated in the DF-particle-exposed cells (i.e., the response was more widespread compared with the HFO-particle-treated cells on all “omic”-levels; p<0.001, Figs 1 and 3 and S5 Fig). The most significantly regulated genes, proteins and metabolites also differed between the DF and HFO (S6 Fig), which shows that the response to emissions of each type of fuel differed quantitatively and qualitatively in both human lung cell lines. A higher regulation alone only proofs a stronger biological reaction onto the deposited PM at the given exposure conditions (i.e. 4 h exposure at a deposition dose below measurable cytotoxicity) and does not necessarily indicate a higher toxicity or risk of disease.

Further conclusions can be drawn from a specific biological pathway analysis. Pro-inflammatory signaling, chemical response (such as xenobiotic metabolism) and oxidative stress pathways were indicated by the regulated genes (Fig 3 and S6 Fig)[19]. The HFO particles specifically induced the transcription of primary and secondary inflammation markers (IL-8, IL-6 and IL-1), and both fuel types affected the cytokines CSF3, CXCL1, and CXCL2. Considering xenobiotic metabolism, CYP1A1 (PAH metabolism) was induced by exposure to HFO particles (which corresponds to the higher PAH concentrations in HFO PM), whereas the DF particles affected other cytochromes (CYP3A4 and CYP17A1) and the carbosulphotransferase CHST6 (Fig 3).

In addition to these, in the context of aerosol exposure well-known pathways [19, 48], we searched for other cellular responses undergoing modulation. A meta-analysis combining the proteome and transcriptome data was performed to examine the significant enrichment of gene ontology (GO) terms. The results indicate that the HFO and DF particle effects were distinct (Fig 3 and, in more detail, S7 Fig). Particles from both fuels induced effects on cell motility, the cellular stress response, the response to organic chemicals, proliferation and cell death (Fig 3 and S7 Fig). Genes and proteins associated with vesicle transport pathways were enriched, which might be connected to the endocytosis of diesel particulate matter.

The pathways specifically regulated by DF particle exposure included the general translation pathway (Fig 3, S7 Fig and S2 Table). The translational elongation, RNA-processing and ribosome translation pathways were down-regulated, whereas the pathways that affect chromatin organisation and modification were up-regulated. The down-regulated pathways included histone acetylation, which may result in DF particle-induced epigenetic changes. Other pathways modulated by DF particles were involved in processes such as cell junction organisation and cell adhesion. Pathways such as the energy metabolism, cell junction and cell adhesion were clearly affected in both cell lines when assessed using transcriptomics and proteomics but differed in the direction of regulation (Table 1, S2 Table and Fig 3), which indicates a time-delayed reaction in the cell. Exposure to DF particles induced mitochondria-associated genes and proteins, which indicates that mitochondrial stress was induced, whereas the HFO particles did not yield this response.

**Table 1:** Summary of the main HFO- and DF-particle exposure effects.

Effect	HFO	DF
Pro-inflammatory signaling	↑	-
Oxidative stress	↑	-
Cell homeostasis	↑	-
Response to chemicals	↑	↓↑
Cellular stress response	↑	↑
Motility	↑	↑
Endocytosis	↑	↑
Cellular signalling	MAPK, TGF beta, PDGF, EGF, GPCR	ID, kinase cascade
Energy metabolism	-	↓↑^x^
Protein synthesis	-	↓
Protein degradation	-	↑
RNA metabolism	-	↓
Chromatin modifications	-	↑
Cell junction and adhesion	-	↓↑*

The arrows indicate the direction of regulation for cellular functions derived from the most statistically significant enriched Gene Ontology terms from the transcriptome, proteome, and metabolome (details in S2 Table).

^x^ BEAS-2B up, A549 down

* BEAS-2B down, A549 up

Pathways specifically regulated by the HFO particles include the homeostasis, oxidative stress and inflammatory response pathways, whereas the metabolic and biosynthetic processes were slightly down-regulated (Fig 3 and S2 Table).

Interestingly, the proteomics data reveal a direct induction of cell-cell interaction remodelling, whereas the transcriptomics data show a down-regulation of similar GO terms. This finding can be explained by assuming an immediate response of the proteome e.g. by stabilizing the already synthesized proteins, while the transcriptome shows the shut-down of the system in a time-delayed response. Although 40% of the observed protein regulation can be explained by the changes in mRNA abundance, most of the changes indicate other modes of regulation. Protein can be degraded in direct response to PM exposure, and translation or transcription may be too slow to change the protein concentrations after 4 h of exposure [49].

The metabolome analyses supported the finding that biosynthetic and protein synthesis processes were down-regulated in the DF particle-treated cells. ATP-binding cassette transporters, which are involved in actively transporting biomolecules across membranes, were also affected (S2 Table). Further information supporting the inhibition of biosynthetic activities includes the negatively affected metabolites secreted by the cells (Fig 3). The pathways affected by HFO particle exposure include glycolysis and pyrimidine metabolism. Glycolysis is a pathway that is typically altered during inflammation and is generally increased in cells under inflammatory conditions [50].

Glucose flux into lactic acid through glycolysis was significantly reduced (p<0.05) in cells treated with DF particles (Fig 3 and S9 Fig). Mammalian cells oxidise glucose and glutamine in the TCA cycle to produce NADH/H^+^, which is re-oxidised in the respiratory chain to produce ATP. DF exposure strongly decreases the levels of relative glucose oxidation in the TCA cycle compared with HFO, as reflected by the significantly lower levels of labelled citric acid (p<0.001; ratio data: Fig 3). Simultaneously, we observed an increase in glucose-derived carbon flux into glycine (Fig 3); enhanced glycine metabolism has previously been associated with tumourigenesis in lung cancer [51]. These observations suggest a lower ATP production and, hence, lower available energy compared with HFO. Increased carbon flux into glycine is directly linked to the increased transformation of hydroxymethyl groups through one-carbon metabolism. The latter is essential for DNA synthesis and repair.

## Conclusions

We assessed human lung cell responses to ship exhaust particles. A unique combination of extensive chemical and physical aerosol characterization and multiple omics analysis was used to generate a broad overview on cellular mechanisms affected by shipping particles and to identify possibly harmful constituents of two types of ship exhaust aerosols. While not providing a classical toxicological risk assessment, which would require the testing of multiple doses and time-points, this study rather gives a comprehensive picture on the cellular responses to ship exhaust particles after short-term exposure, which should be used as starting point for more mechanistic studies. Although the HFO particles deposited in the ALI system were about equal in mass, higher in number and contained a large excess of toxic compounds, DF particle exposure induced a broader biological reaction in the human lung cells (BEAS-2B and A549) on all investigated "omic" levels. As discussed, a stronger affected cell metabolism is not an adverse effect *per se*, but it holds a higher risk of disturbance of normal cell functions. Within known pathways, such as pro-inflammatory signaling, oxidative stress and xenobiotic metabolism, the levels of certain well-known indicators (e.g., IL-1/6/8 and CYP1A1) surged following HFO particle exposure. In contrast, DF particles strongly affected basic cellular functions (energy and protein metabolism) and mechanisms little yet known to be affected by aerosol treatment, such as mRNA processing and chromatin modification.

The obtained results also suggest formulating specific hypotheses and are motivating further experiments to proof or disproof those. In this context the role of freshly formed “elemental carbon, EC” fractions and the influence of organic compounds on the biological activity should be investigated. The relatively large EC fraction in DF exhaust is one of the prominent differences between the two particle types. The chemical and physical surface properties of freshly formed EC fractions might be of relevance here. Laboratory experiments using e.g. combustion aerosol standard generator (CAST,[52]), which allows to generate fresh combustion particles with adjustable EC/OC ratios, are currently under preparation.

There is no doubt that the carcinogenic emissions from HFO-operated vessels need to be minimized and HFOs should be replaced by refined modern DF (at least if no flue gas cleaning systems are installed). HFO emissions contain among other constituents high concentrations of toxic metals (V, Ni etc.) and polycyclic aromatic hydrocarbons. However, also emission of diesel engines operated with refined DF, are known to be toxic and carcinogenic, although the toxicant concentrations are much lower [8] than in HFO emissions. Consequently the implementation of emission reduction measures for land-based diesel engines started decades ago (e.g. with sulfur-reduced fuels) [18] and current efforts are directed towards the reduction of particle emissions from diesel automobiles. Due to the substantial contribution of ship emissions to global pollution, ship emissions are the next logical target for improving air quality worldwide, particularly in coastal regions and harbour cities. In this context our findings on the biological effects of HFO and DF ship diesel emissions can contribute to the current debate about the reduction measures to be implemented for shipping. The results from this study provide the information that at comparable lung deposition doses the acute biological activity of particles of ship emissions from DF fuelled ships is not less relevant than the activity of HFO emission particles. This supports the suggestion that a general reduction of the PM emissions (not the SO_2_ emission) from shipping in harbours and the vicinity of the coast should be implemented for both, HFO- and DF-operated ships. Efficient particle filter technology (e.g., electrostatic precipitation or bag-filtration) is available. From a regulatory perspective, the next step should be the introduction of legal emissions limits for respirable PM (e.g. PM 2.5, in [mg/m^3^]) from ship emissions [29].

## Supporting Information

S1 Fig Sampling setup. (A) Simplified scheme of the sampling and measurement setup. DR = dilution ratio, TC = temperature control, T = temperature measurement, P = pressure meter. (B) Detailed setup of the used sampling train with porous tube and ejector diluter units. (C) Properties of the used diesel fuel (DF) and heavy fuel oil (HFO). Most noticeable are the high viscosity and high sulfur content of HFO compared with distillate fuels like EN 590 diesel. (D) Experimental engine parameters. The engine is a single cylinder engine with common rail injection system representing state of the art medium speed marine diesel engines. The dual fuel system allows operation with both distillate and residual fuels.
(EPS)

S2 Fig Air-Liquid-Interface (ALI) exposure. HICE exposure system: the left part shows the data acquisition and control unit for the mass flow controllers, humidity and temperature. The exposure unit in the right part contains three Vitrocell modules and is thermostated to 37°C. Each module has six positions for cell exposure to either complete or filtered aerosol for gas phase referencing. The flow through each of the exposure positions is individually controlled by a mass flow controller (lower left) Cell exposure: the aerosol passes through the aerosol inlet and is streaming directly over the cell cultures.
(EPS)

S3 Fig Particle dosing and morphology. (A) Cell viability at DF and HFO aerosol particle dose. A549 cells were exposed for 4h to 1:40 diluted DF or 1:100 diluted HFO. Directly after exposure, cell viability was measured by reduction of Alamar Blue and compared to cells exposed to the filtered aerosols. Reported are the means relative to filtered aerosol ± SD from 3 (HFO) or 2 (DF) independent experiments. As requested for the further ‘omics study, the viability is not impaired by the DF or HFO particle exposure. (B) Size dependent deposited dose of DF and HFO particles (left ordinate) as well as deposition probability (W, right ordinate) calculated according to Comouth et al. (1) for a size dependent density profile. (C) Mass dose of DF and HFO particles deposited per cell area. Data are estimated from gravimetric filter samples (case 1, 2) and from electrical low pressure impactor (ELPI) size distributions (case 3, 4). Calculations are performed assuming a constant deposition probability of W = 1.5% for all particle diameters (case 1, 2). For comparison, calculations are performed additionally using the size dependent probability Wρ(D) based on Comouth et al. (*31*) and a particle density based on a mass-mobility relationship for DF and HFO (case 3, 4). In all cases the deposited PM dose is about a factor 2 higher for the HFO case. d-g, TEM images of diesel fuel exposure aerosol particles. The typical soot agglomerate structure (D,E) and the layered graphitic structure (F,G) is typical for rather pure, elemental carbon containing soot. (H-L), TEM images of heavy fuel oil exposure aerosol particles. The often much smaller particles consist of heavier elements (black speckles) and tarry substance (crusted appearance). The HFO-EA soot particles have a more amorphous structure than the diesel fuel soot (J).
(EPS)

S4 Fig Compounds in particulate matter. (A) Exemplary sum-parameters and compound-class data for exposure aerosol (EA) particulate matter for HFO-EA and DF-EA. Particular abundance and statistic parameters’ ratios (a), absolute concentrations (b) and statistic parameters on the sample complexity (c) reveal a substantial complexity of the organic-chemical composition of the particulate matter. ^1^EC/OC coupled to SPI, ^2^EC/OC coupled to REMPI, ^3^EC/OC-analysis (thermal-optical method), ^4^AMS, ^5^Filter weighing. ^6^Aethalometer, ^7^Comprehensive two-dimensional gas chromatography/Time-of-flight mass spectrometry, ^8^Fourier-Transform Ion Cyclotron Resonance Mass Spectrometry with atmospheric chemical ionization, ^9^Fourier-Transform Ion Cyclotron Resonance Mass Spectrometry with electrospray ionization, ^10^Thermal desorption/direct derivatization gas chromatography/Mass spectrometry, ^11^GC/MS. (B) Elemental analysis of the particulate matter. Exemplary concentrations-ratios (HFO-EA- over DF-EA-particles) of elements (left). Absolute concentrations of the species in the HFO-EA- (red bars) and DF-EA-particles (blue bars) are also shown (right). Method: ICP-AES. (C) Exemplary concentration-ratios (HFO-EA- over DF-EA-particles) of polycyclic aromatic hydrocarbons (PAH) (left). Absolute concentrations of the species in the HFO-EA- (red bars) and DF-EA-particles (blue bars) are also shown (right). The larger the PAH-structure, the stronger is the prevalence of the compound in the HFO-EA-particles. Methods: ^1^Thermal desorption/derivatization gas chromatography/Mass spectrometry, ^2^Gas chromatography/mass spectrometry, ^3^Liquid chromatography/Tandem mass spectrometry. (D) Exemplary concentration-ratios (HFO-EA- over DF-EA-particles) of aliphatic hydrocarbons (left). Absolute concentrations of the species in the HFO-EA- (red bars) and DF-EA-particles (blue bars) are also shown (right). The same behaviour as in the PAH compound class is observed: The larger the aliphatic-structure, the stronger is the prevalence of the compound in the HFO-EA-particles.
(EPS)

S5 Fig DF regulates more transcripts, proteins and metabolites than HFO. (A-C) Comparison of regulation magnitude and regulation significance (obtained with a two-tailed t-Student’s t-test on the replicate measurements). Mean of log2 fold change aerosol/filtered is plotted vs. -log10 p-value of complete datasets of transcriptome in BEAS-2B cells (A), proteome (B) and metabolome (C) in A549 cells for DF and HFO. (D-F), Comparison of regulation magnitude and abundance of regulated transcripts, proteins or metabolites. Mean of log_2_ fold change aerosol/filtered is plotted vs. mean of log_10_ fold intensity of complete datasets of transcriptome (D), proteome (E) and metabolome (F) for DF and HFO.
(EPS)

S6 Fig Cellular responses to DF and HFO differ qualitatively. (A-C) Distinct patterns of regulation of DF and HFO. Hierarchical clustering of highest regulated entities of each omic approach: transcriptomics (A) (BEAS-2B), proteomics (B) and metabolomics (C) (A549). (D,E) Pathways known to be affected by diesel particle exposure. Transcriptome pathway analysis was performed using 1.5-fold regulated genes. Typical PM-influenced pathways were selected and according gene regulation were clustered hierarchically. Apoptosis (D, pro- and anti-apoptotic genes), Oxidative stress (E).
(EPS)

S7 Fig Meta-analysis of gene ontology-terms in the proteomic and transcriptomic measurement of DF and HFO particle-treated samples. Significantly regulated proteins in A549 cells were determined using 10% of lowest and 10% of highest log2 fold change in the ratio Aerosol/Gas and a cut-off of—log10(p-value) >1 for 3 replicates. According to the high identification number, significantly regulated transcripts in BEAS-2B cells were determined using 5% of lowest and 5% of highest log2 fold change of Aerosol/Gas and a cut-off of—log10(p-value) >1 for 3 replicates. GO term analysis was performed using David Tool. The p-values of GO-terms were z-transformed, hierarchically clustered, and plotted as a heat map.
(EPS)

S8 Fig DF- and HFO-particles disrupt lung epithelial integrity. (A) Histopathology of HFO-/DF-particle treated NHBE cells. Light microscopy histological analysis of sections of the NHBE cultures treated with PBS (control), and (B) HFO, (C) DF and (D) CB120 at a dose 150 μg/cm^2^ for 24 h. Hematoxylin and eosin staining, scale bar = 50 μm. (E) TEM micrographs of HFO- and (F) DF-particle treated NHBE cells. Ribosome agglomeration in cells of the NHBE cultures after 24 h incubation at a dose 150 μg/cm^2^; n = 5. Scale bar = 2 μm.
(EPS)

S9 Fig Secreted metabolites and metabolomic flux analysis. Metabolism of U-^13^C-Glucose through central carbon metabolism in A549 cells. Reduced model of central carbon metabolism, with labeled atom transition marked for selected metabolites. Red circles = ^13^C labeled carbon; Blue circles = ^13^C labeled carbon from Malic Enzyme activity; White circles = ^12^C unlabeled carbon. Selected Secreted Metabolite Ratios. Selected metabolites were measured through GC/MS analysis of cellular medium post exposure. Values shown are the ratios of unfiltered treatments to filtered treatments for each fuel type during three replicates. Metabolic flux measurements based on ^13^C-labeled glucose. Filtered and unfiltered aerosol samples were analyzed separately.
(EPS)

S10 Fig Exemplary light microscopic image of a confluent A459 cell layer. 4x10^5^ A549 cells were seeded into a 24mm trans-well insert. After 24h and just before ALI-exposure, confluence was checked by light microscopy.
(TIF)

S1 Table Chemical Analytics of Ship Exhaust Particles. (XLSX)

S2 Table Biological Responses to Ship Exhaust Particles. (XLSX)

S1 Text Materials and Methods. (DOCX)

## References

[1] Pope CA 3rd, Dockery DW. Air pollution and life expectancy in China and beyond. Proc Natl Acad Sci USA. 2013;110(32):12861–2. 10.1073/pnas.1310925110 23847200 PMC3740834

[2] Chen Y, Ebenstein A, Greenstone M, Li H. Evidence on the impact of sustained exposure to air pollution on life expectancy from China's Huai River policy. Proc Natl Acad Sci USA. 2013;110(32):12936–41. 10.1073/pnas.1300018110 23836630 PMC3740827

[3] Benbrahim-Tallaa L, Baan RA, Grosse Y, Lauby-Secretan B, El Ghissassi F, Bouvard V, et al Carcinogenicity of diesel-engine and gasoline-engine exhausts and some nitroarenes. Lancet oncol. 2012;13(7):663–4. 22946126 10.1016/s1470-2045(12)70280-2

[4] Dalsoren SB, Eide MS, Endresen O, Mjelde A, Gravir G, Isaksen ISA. Update on emissions and environmental impacts from the international fleet of ships: the contribution from major ship types and ports. Atmos Chem Phys. 2009;9:2171–94.

[5] Matthias V, Bewersdorff I, Aulinger A, Quante M. The contribution of ship emissions to air pollution in the North Sea regions. Environ pollut. 2010;158(6):2241–50. 10.1016/j.envpol.2010.02.013 20226578

[6] Ault AP, Moore MJ, Furutani H, Prather KA. Impact of emissions from the Los Angeles port region on San Diego air quality during regional transport events. Environ Sci Technol. 2009;43(10):3500–6. 19544846 10.1021/es8018918

[7] Poplawski K, Setton E, McEwen B, Hrebenyk D, Graham M, Keller P. Estimation and assesment of cruise ship emissions in Victoria, BC, Canada. Atmos Environ. 2011;45:824–33.

[8] Corbett JJ, Winebrake JJ, Green EH, Kasibhatla P, Eyring V, Lauer A. Mortality from ship emissions: a global assessment. Environ Sci Technol. 2007;41(24):8512–8. 18200887 10.1021/es071686z

[9] EPA. Diesel Boats and Ships. Available: http://www.epa.gov/otaq/marine.htm. Accessed March 2014) 2014.

[10] Lack DA, Cappa CD, Langridge J, Bahreini R, Buffaloe G, Brock C, et al Impact of fuel quality regulation and speed reductions on shipping emissions: implications for climate and air quality. Environ Sci Technol. 2011;45(20):9052–60. 10.1021/es2013424 21910443

[11] Blatcher DJ, Eames I. Compliance of Royal Naval ships with nitrogen oxide emissions legislation. Marine pollution bulletin. 2013;74:10–8. 10.1016/j.marpolbul.2013.07.010 23906471

[12] Winebrake JJ, Corbett JJ, Green EH, Lauer A, Eyring V. Mitigating the health impacts of pollution from oceangoing shipping: an assessment of low-sulfur fuel mandates. Environ Sci Technol. 2009;43(13):4776–82. 19673264 10.1021/es803224q

[13] Borrell Fontelles J, Straw J. Directive 2005/33/EC of the Europen Parliament and the council. OJEU. 2005;L191/59(22 7 2005).

[14] Khan MY, Giordano M, Gutierrez J, Welch WA, Asa-Awuku A, Miller JW, et al Benefits of two mitigation strategies for container vessels: cleaner engines and cleaner fuels. Environ Sci Technol. 2012;46(9):5049–56. 10.1021/es2043646 22468877

[15] Gaetjens. http://smm-hamburg.com/fileadmin/img/content/programme/downloads/programmpunkte_de/491_7351_gaetjens.pdf 2012.

[16] Eyring V, Isaksen ISA, Berntsen T, Collins WJ, Corbett JJ, Endresen O, et al Transport impacts on atmosphere and climate: Shipping. Atmospheric Environment. 2010;44(37):4735–71. 10.1016/j.atmosenv.2009.04.059 PMC7110594 32288556

[17] Eyring V, Köhler HW, van Aardenne J, Lauer A. Emissions from international shipping. J Geophys Tes. 2005;110:D17305.

[18] Lloyd AC, Cackette TA. Diesel engines: environmental impact and control. J Air & Waste Manag Assoc. 2001;51(6):809–47. 11417675 10.1080/10473289.2001.10464315

[19] Schwarze PE, Totlandsdal AI, Lag M, Refsnes M, Holme JA, Ovrevik J. Inflammation-related effects of diesel engine exhaust particles: studies on lung cells in vitro. BioMed research international. 2013;(685142):1–13. 10.1155/2013/685142 23509760 PMC3586454

[20] Holder AL, Lucas D, Goth-Goldstein R, Koshland CP. Cellular response to diesel exhaust particles strongly depends on the exposure method. Toxicol Sci: an official journal of the Society of Toxicology. 2008;103(1):108–15. 10.1093/toxsci/kfn014 18227103

[21] Paur H-R, Cassee F, Teeguarden J, Fissan H, Diabate S, Aufderheide M, et al In-vitro cell exposure studies for the assessment of nanoparticle toxicity in the lung—A dialog between aerosol science and biology. J Aerosol Sci. 2011;42(10):668–92. 10.1016/j.jaerosci.2011.06.005

[22] Tsukue N, Okumura H, Ito T, Sugiyama G, Nakajima T. Toxicological evaluation of diesel emissions on A549 cells. Toxicol in vitro. 2010;24(2):363–9. 10.1016/j.tiv.2009.11.004 19900534

[23] Cooney DJ, Hickey AJ. Cellular response to the deposition of diesel exhaust particle aerosols onto human lung cells grown at the air-liquid interface by inertial impaction. Toxicol in vitro. 2011;25(8):1953–65. 10.1016/j.tiv.2011.06.019 21756993

[24] Oostingh GJ, Papaioannou E, Chasapidis L, Akritidis T, Konstandopoulos AG, Duschl A. Development of an on-line exposure system to determine freshly produced diesel engine emission-induced cellular effects. Toxicol in vitro. 2013;27(6):1746–52. 10.1016/j.tiv.2013.04.016 23684770

[25] Kooter IM, Alblas MJ, Jedynska AD, Steenhof M, Houtzager MM, Ras M. Alveolar epithelial cells (A549) exposed at the air-liquid interface to diesel exhaust: First study in TNO's powertrain test center. Toxicol in vitro. 2013;27(8):2342–9. 10.1016/j.tiv.2013.10.007 24161370

[26] Adam TW, Chirico R, Clairotte M, Elsasser M, Manfredi U, Martini G, et al Application of modern online instrumentation for chemical analysis of gas and particulate phases of exhaust at the European Commission heavy-duty vehicle emission laboratory. Anal Chem. 2011;83(1):67–76. Epub 2010/12/04. 10.1021/ac101859u 21126058

[27] Mueller D, Uibel S, Takemura M, Klingelhoefer D, Groneberg DA. Ships, ports and particulate air pollution—an analysis of recent studies. J Occup Med Toxicol. 2011;6:31 10.1186/1745-6673-6-31 22141925 PMC3244961

[28] Cooper J. Exhaust emissions from ships at berth. Atmos Environ. 2003;37:3817–30.

[29] Winnes H, Fridell E. Particle emissions from ships: dependence on fuel type. J Air & Waste Manag Assoc. 2009;59(12):1391–8. 20066904 10.3155/1047-3289.59.12.1391

[30] Reddel RR, Ke Y, Gerwin BI, McMenamin MG, Lechner JF, Su RT, et al Transformation of human bronchial epithelial cells by infection with SV40 or adenovirus-12 SV40 hybrid virus, or transfection via strontium phosphate coprecipitation with a plasmid containing SV40 early region genes. Cancer Res. 1988;48(7):1904–9. 2450641

[31] Kooter IM, Alblas MJ, Jedynska AD, Steenhof M, Houtzager MM, van Ras M. Alveolar epithelial cells (A549) exposed at the air-liquid interface to diesel exhaust: First study in TNO's powertrain test center. Toxicol in vitro. 2013;27(8):2342–9. 10.1016/j.tiv.2013.10.007 24161370

[32] Steinritz D, Mohle N, Pohl C, Papritz M, Stenger B, Schmidt A, et al Use of the Cultex(R) Radial Flow System as an in vitro exposure method to assess acute pulmonary toxicity of fine dusts and nanoparticles with special focus on the intra- and inter-laboratory reproducibility. Chemico-biol Int. 2013;206(3):479–90. 10.1016/j.cbi.2013.05.001 23669118

[33] Herzog F, Clift MJ, Piccapietra F, Behra R, Schmid O, Petri-Fink A, et al Exposure of silver-nanoparticles and silver-ions to lung cells in vitro at the air-liquid interface. Part Fibre Toxicol. 2013;10:11 10.1186/1743-8977-10-11 23557437 PMC3639923

[34] Persoz C, Achard S, Momas I, Seta N. Inflammatory response modulation of airway epithelial cells exposed to formaldehyde. Toxicol Lett. 2012;211(2):159–63. 10.1016/j.toxlet.2012.03.799 22484645

[35] Baber O, Jang M, Barber D, Powers K. Amorphous silica coatings on magnetic nanoparticles enhance stability and reduce toxicity to in vitro BEAS-2B cells. Inhal Toxicol 2011;23(9):532–43. 10.3109/08958378.2011.592869 21819260

[36] Diabate S, Mulhopt S, Paur HR, Krug HF. The response of a co-culture lung model to fine and ultrafine particles of incinerator fly ash at the air-liquid interface. Atla-Altern Lab Anim. 2008;36(3):285–98. 18662093 10.1177/026119290803600306

[37] Patel MM, Chillrud SN, Deepti KC, Ross JM, Kinney PL. Traffic-related air pollutants and exhaled markers of airway inflammation and oxidative stress in New York City adolescents. Environ Res. 2013;121:71–8. 10.1016/j.envres.2012.10.012 23177171 PMC3577992

[38] Comouth A, Saathoff H, Naumann K-H, Muelhopt S, Paur H-R, Leisner T. Modelling and measurement of particle deposition for cell exposure at the air—liquid interface. J Aerosol Sci. 2013;63(0):103–14. 10.1016/j.jaerosci.2013.04.009

[39] Karthikeyan S, Thomson EM, Kumarathasan P, Guenette J, Rosenblatt D, Chan T, et al Nitrogen dioxide and ultrafine particles dominate the biological effects of inhaled diesel exhaust treated by a catalyzed diesel particulate filter. Toxicol Sci. 2013;135(2):437–50. 10.1093/toxsci/kft162 23897985

[40] Ghio AJ, Dailey LA, Soukup JM, Stonehuerner J, Richards JH, Devlin RB. Growth of human bronchial epithelial cells at an air-liquid interface alters the response to particle exposure. Part Fibre Toxicol. 2013;10(1):25 10.1186/1743-8977-10-25 23800224 PMC3750262

[41] Dept. of Health and Human Services. NIOHS pocket guide to chemical hazards. Barsan M, editor. Cincinnati Ohio: NIOHS publications; 2007.

[42] Pagels J, Khalizov AF, McMurry PH, Zhang RY. Processing of Soot by Controlled Sulphuric Acid and Water Condensation: Mass and Mobility Relationship. Aerosol Sci Technol. 2009;43(7):629–40. 10.1080/02786820902810685

[43] Park K, Cao F, Kittelson DB, McMurry PH. Relationship between particle mass and mobility for diesel exhaust particles. Environ Sci Technol. 2003;37(3):577–83. 10.1021/es025960v 12630475

[44] Ferron GA, Upadhyay S, Zimmermann R, Karg E. Model of the Deposition of Aerosol Particles in the Respiratory Tract of the Rat. II. Hygroscopic Particle Deposition. J Aerosol Med Pulm Drug Deliv. 2013;26(2):101–19. 10.1089/jamp.2011.0965 23550602

[45] Karg E, Ferron GA. The hygroscopic particle lung deposition model Neuherberg / Munich: Helmholtz Zentrum München; 2014 [cited 2014]. Available: http://www.helmholtz-muenchen.de/en/neu-cma/research/facilities/lung-deposition-model/index.html.

[46] Wehner B, Birmili W, Gnauk T, Wiedensohler A. Particle number size distributions in a street canyon and their transformation into the urban-air background: measurements and a simple model study. Atmos Environ. 2002;36(13):2215–23.

[47] Vignati E, Berkowicz R, Palmgren F, Lyck E, Hummelshoj P. Transformation of size distributions of emitted particles in streets. Sci Total Environ. 1999;235(1–3):37–49. 10535125

[48] Oeder S, Jorres RA, Weichenmeier I, Pusch G, Schober W, Pfab F, et al Airborne indoor particles from schools are more toxic than outdoor particles. Am J Respir Cell Mol Biol. 2012;47(5):575–82. 10.1165/rcmb.2012-0139OC 22904196

[49] Schwanhausser B, Busse D, Li N, Dittmar G, Schuchhardt J, Wolf J, et al Global quantification of mammalian gene expression control. Nature. 2011;473(7347):337–42. 10.1038/nature10098 21593866

[50] Palsson-McDermott EM, O'Neill LA. The Warburg effect then and now: from cancer to inflammatory diseases. BioEssays. 2013;35(11):965–73. 10.1002/bies.201300084 24115022

[51] Zhang WC, Shyh-Chang N, Yang H, Rai A, Umashankar S, Ma S, et al Glycine decarboxylase activity drives non-small cell lung cancer tumor-initiating cells and tumorigenesis. Cell. 2012;148(1–2):259–72. 10.1016/j.cell.2011.11.050 22225612

[52] Mueller L, Jakobi G, Orasche J, Karg E, Sklorz M, Abbaszade G, et al Online determination of polycyclic aromatic hydrocarbon formation from a flame soot generator. Anal Bioanal Chem. 2015 10.1007/s00216-015-8549-x 25711989

