# Supplementary material for: Particulate Matter from Both Heavy Fuel Oil and Diesel Fuel Shipping Emissions Show Strong Biological Effects on Human Lung Cells at Realistic and Comparable In Vitro Exposure Conditions
Source: PLoS One. 2015 Jun 3;10(6):e0126536. doi: 10.1371/journal.pone.0126536 (PMC4454644; doi:10.1371/journal.pone.0126536)
Supplement: S1 Text — (DOCX) [file pone.0126536.s013.docx]

**Text S1. Materials and Methods**

**Engine**

A four-stroke single-cylinder direct-injected diesel engine was used. Details are summarized in Fig. S1d. Heavy fuel oil HFO 180 was used as a representative fuel for ship operation outside of sulfur emission-control-areas (SECAs). Distillate fuel according to DIN EN 590 was used as a reference. Properties and measured data of both fuel types are listed in Fig. S1c. The engine ran at four different operating points: 100 %, 75 %, 50 %, 25 % load at nominal speed of 1,500 min^-1^. The duration of each operation point was set in accordance to their weighting factors as described in ISO 8178-4 E2. The total cycle duration was 2 hours, which was run twice in a row.

**Sampling**

Aerosol samples from the exhaust pipe were taken with a diluting aerosol sampling system (Venacontra, DAS, Finland). Depending on the desired concentration range dilution was carried out in 2-4 stages (Fig. S1) with clean and particle free compressed air at room temperature. The primary dilution was carried out using a porous tube diluter ([Lyyranen et al. 2004](#_ENREF_9)).

Four parallel **PM_2.5_ filter samples** were collected with a modified speciation sampler (Rupprecht & Patashnik 2300, Thermo Scientific, Waltham, USA) simultaneously and under the same dilution ratio as the ALI exposure. PM samples were collected on quartz fiber filters (QFF, T293, Munktell, Sweden) and PTFE membrane filters (PFF, Zefluor 1 µm, Pall, USA).

**Carbonyl compounds** (CCs) from the gas phase were sampled by derivatisation with 2,4-dinitrophenylhydrazine (DNPH) cartridges.

The samples for **electron microscopy** were collected on perforated carbon substrates supported by copper mesh (Agar Scientific). The morphology of the particles was studied by transmission electron microscopy (TEM) (JEM 2100F, JEOL Ltd., Japan). The elemental composition of the particles was analyzed using energy dispersive X-ray spectroscopy (EDS, NORAN System 7, Thermo Scientific, USA).

**On-line methods**

**Gaseous emissions** were analyzed in real time from the undiluted raw gas using commercial gas monitors (AVL CEB II) at 1 Hz sampling frequency.

**Photoionization Time-of-Flight (TOF) mass spectrometry** was carried out by two independent systems. Firstly, VUV light (126 nm, 9.8 eV) from a continuous-wave excimer lamp served as an ionization source in an orthogonal extraction TOF-spectrometer operating at 60 kHz repetition rate (Tofwerk C-TOF). In contrast, the second TOF-instrument (Kaesdorf CTF10) utilized intense, pulsed laser radiation (≈10^7^ W/cm^2^) in the UV range (266 nm, 4.66 eV, 4th harmonic of NdYAG,) for resonance enhanced multiphoton ionization (REMPI).

**A High-resolution Time-of-Flight Aerosol Mass Spectrometer** (HR-TOF-AMS, Aerodyne Research Inc., USA) was used to measure sub-micrometer non-refractory particles. Diluted exhaust particles were sampled directly and focused into a tight particle beam, which hits a conical surface, heated to 600 °C, where the non-refractory components of the particles were evaporated and continuously ionized by electrons of 70 eV. Ions are analyzed by a fast reflectron TOF-system. Mass spectra were recorded every 20 seconds.

**Black Carbon (BC)** measurements were carried out with a 7-wavelength Aethalometer (AE33, MAGEE Scientific), described in detail by Arnott et al. ([Arnott et al. 2005](#_ENREF_2)).

**Particle size distribution and number concentration** were measured in parallel by a Scanning Mobility Particle Sizer (SMPS, TSI, USA) ([Reischl 1991](#_ENREF_15)) and an Electrical Low Pressure Impactor (ELPI, Dekati, Finland) ([Keskinen et al. 1992](#_ENREF_7)). The SMPS was set for a particle size range of 15 nm to 638 nm and the ELPI for an aerodynamic size range of 14 nm to 6.3 µm. Furthermore, particle number concentrations were continuously monitored with a Condensation Particle Counter (CPC, TSI, USA) ([Wiedensohler 1997](#_ENREF_18)). Deposited dose and size distribution inside the ALI was calculated from the measured data in the diluted aerosol, overlaid by the cut-off function of the PM_2.5_ ALI-pre-impactor.

**Off-line methods**

**EC/OC analyses** were carried out by a thermal-optical carbon analyzer (Desert Research Institute Model 2001A) applying the *Improve A* protocol ([Chow et al. 2007](#_ENREF_3)). The analyzer was coupled with a time-of-flight mass spectrometer using single photon and resonance-enhanced multi-photon ionization (SPI and REMPI) ([Grabowsky et al. 2011](#_ENREF_6)).

**In-situ derivatisation and thermal desorption coupled to gas chromatography followed by a time-of-flight mass spectrometer** (Leco, USA) was applied to analyze organic compounds on sampled particles ([Orasche et al. 2011](#_ENREF_12)).

**Carbonyl compounds (CCs)** were eluted from the cartridge with 1ml acetonitrile and analyzed by GC-MS (Shimadzu GCMS-QP2010).

**Extraction of particulate samples for the** analyses of organic compounds were performed from QFF filters. The filters were extracted twice in an ultrasonic bath with 25 ml methanol/dichloromethane (50/50, v/v), respectively. For analysis of nitro-PAH and methyl-PAH solid-phase extraction (SPE) of one sixth of the whole extract was performed.

**For the determination of nitro-PAH** a method based on the post-column reduction of nitro-compounds followed by fluorescence detection of the resulting amino-PAH was used ([Schauer et al. 2004](#_ENREF_16)).

**Methylated PAHs** were analyzed from the first eluates of the solid phase extraction by GC-MS/MS. 1 µl solution with added internal standard was injected splitless into a PolarisQ (Thermo Scientific, Dreieich, Germany) Ion-Trap GC-MS injector at 280 °C. 16-methylated PAH including retene were quantified.

**Two-dimensional GCxGC-ToF-MS** analysis of the filter aerosols was carried out using an Agilent 6890 gas chromatograph equipped with a LECO Pegasus 4D option, including a Pegasus III TOF-MS (LECO, ST. Joseph, MI, USA) using helium as carrier gas ([Welthagen et al. 2003](#_ENREF_17)).

Analyses by **Electrospray-Ionization-Mass-Spectrometry (ESI-FTICR-MS)** were carried out by a solariX FT-ICR mass spectrometer (Bruker Daltonik GmbH, Bremen, Germany) equipped with a 7 Tesla superconducting magnet (Bruker Biospin, Wissembourg, France) in positive (+) ion mode. The mass spectrometer was tuned for a mass-to-charge-interval of 150 to 1000 amu with a resolution of around 200.000 @ m/z 400.

**Gas chromatography coupled to atmospheric pressure chemical ionization mass spectrometry (GC-APCI-FTICR-MS)** was performed using a CP 3800 gas chromatograph (Agilent, Palo Alto, CA, USA) equipped with a programmable temperature vaporizing injector (model 1079) hyphenated to an Apex Qe Series II FTMS system (Bruker Daltonics, Billerica, MA, USA / Bruker Daltonics GmbH, Bremen, Germany). The APCI source and the FT-ICR MS were operated in positive (+) ion mode.

**Elements** from the particulate samples were analyzed using inductively coupled plasma (ICP) atomic emission spectrometry (ICP-AES, „Spectro Ciros Vision“ system from SPECTRO Analytical Instruments GmbH & Co. KG, Kleve, Germany).

**Cell culture conditions**

Human lung epithelial cell line A549 (American Type Culture Collection; ATCC® CCL-185™; http://www.lgcstandards-atcc.org/Products/All/CCL-185.aspx) was cultured in RPMI-1640 medium supplemented with 10% (v/v) fetal bovine serum (FBS), 100 U/ml penicillin, 100 mg/ml streptomycin (all from Life Technologies, Darmstadt). Human immortalized bronchial epithelial cells BEAS-2B (ATCC® CRL-9609™; http://www.lgcstandards-atcc.org/Products/All/CRL-9609.aspx) were cultured in Bronchial Epithelial Growth Medium (BEGM, Lonza Inc., Walkersville, MD) supplemented with 100 units/ml penicillin/streptomycin and on plates that were pre-coated with 0.01 mg/ml fibronectin, 0.03 mg/ml bovine collagen Type 1 and 0.01 mg/ml BSA.

**Air-liquid-interface exposure chamber**

To provide reproducible experimental conditions for the exposure of the bioassays at the air-liquid-interface the HICE-Exposure system was applied based on previous work ([Mülhopt et al. 2008](#_ENREF_10)). In this fully automatic system cell cultures grown on porous membrane inserts, which supply the nutrient medium, are exposed directly to diluted aerosol from combustion sources (Fig. S2). The aerosol is humidified by steam injection to 85 % relative humidity and temperature is controlled to 37°C. The flow rate is 100 ml/min.

**Air-liquid interface exposure**

Cells were placed into isolator cups that were previously filled with RPMI 1640 medium (for A549 cells) or bronchial epithelial basal medium (BEBM, Lonza Inc., Walkersville, MD; for BEAS-2B cells), each supplemented with 10 mM HEPES. Cells were then exposed for 4h at the air-liquid-interface (ALI) exposure system (Fig. S2). Each exposure condition was tested 3 times. Aerosol was diluted 1:40 and 1:100 for DF and HFO, respectively. The deposited dose was estimated from the mass concentration data of the fitted particle size distributions for DF and HFO (Fig. S3b).

**Viability test (AlamarBlue®)**

Viability of the cells was tested using Alamar blue ([Kooter et al. 2013](#_ENREF_8)).

**Whole-genome expression analysis**

For the analysis of gene expression on whole-genome level Agilent one-color microarrays were used ([Adam et al. 2011](#_ENREF_1)). Total RNA was extracted using the AllPrep RNA/Protein Kit (Qiagen, Hilden, Germany), purified and Cy3-labeled according to the manufacturers protocol and analyzed on Sure Print G3 Human Gene Expression Microarrays (8x60 K, Agilent, Waldbronn, Germany).

**Stable isotope labeling (SILAC) by D_4_-Lysine and ^13^C_6_-Glucose labeling**

Human A549 lung cells were cultured for 6 passages ([Ong and Mann 2006](#_ENREF_11)) in RPMI-1640 media supplemented with 10% dialyzed FBS (Sigma-Aldrich), 1 × penicillin/streptomycin (Sigma-Aldrich), either 48.67 μg/ml lysine (Sigma-Aldrich) or D_4_-lysine (Lys4, Sigma-Aldrich) to achieve complete labeling of the proteome. The D_4_-lysine labeled cells were seeded 24 h before the experiment in RPMI 1640 media additionally containing 0.0125M ^13^C_6_-Glucose (Cambridge Isotope Laboratories, USA).

**Protein and metabolite extraction from one sample**

Cells were harvested and pipetted into an Eppendorf-tube containing 400 μL of at -20°C chilled HPLC-grade chloroform. Polar and non-polar phases were physically separated. Afterwards 200 µL of the aqueous upper phase containing the polar metabolites was stored at -80^0^C until analysis. The non-polar phase was discarded. The interphase containing the precipitated protein was used for proteomics.

**Proteomics**

Proteins were resuspended in 60 µl of denaturation buffer (6 M urea, 2 M thiourea, 20 mM HEPES, pH 8.0). Labeled and unlabeled samples were mixed 1:1 to get a total amount of 100 μg proteins. Protein quantitation was performed using the Bradford assay. Strong-anion exchange chromatography (SAX) fractionation of peptides was performed as described in Wisniewski et al. ([Wisniewski et al. 2009](#_ENREF_19)).

**Metabolomics**

Metabolite extracts were measured on an Agilent 7890 GC containing a 30 m DB-35MS capillary column. The GC was connected to an Agilent 5975C MS operating in electron ionization (EI) mode at 70 eV. The detector was operated in scan mode with an m/z range of 70 to 800. Derivatisation was carried out by 2,2,2-trifluoro-N-methyl-N-trimethylsilyl-acetamide (Sigma, UK).

**Omics data analysis**

All data analysis was performed with *R* ([R-Core-Team 2012](#_ENREF_14)) and the Bioconductor ([Gentleman et al. 2004](#_ENREF_5)) packages *affy, limma* and *gplots*([Gautier et al. 2004](#_ENREF_4)). Heatmaps were produced by first performing a hierarchical cluster analysis to the log2-fold changes (using the Ward Method) and then plotting the colored values.

**Off-line analyses at the bronchial epithelium model**

Primary normal human bronchial epithelial (NHBE) cells obtained from non-smoking, post-mortem individuals, were used (Lonza, Switzerland). Cells were cultured as previously described ([Prytherch et al. 2011](#_ENREF_13)). The following particles were used: particles from heavy fuel oil (HFO) and diesel fuel (DF) collected on polycarbonate filters (Millipore, UK), carbon black (CB120), Monarch 120 (Cabot, UK) and DQ12 quartz particles (Sigma, UK).

Supplementary References

Adam TW, Chirico R, Clairotte M, Elsasser M, Manfredi U, Martini G, et al. 2011. Application of modern online instrumentation for chemical analysis of gas and particulate phases of exhaust at the european commission heavy-duty vehicle emission laboratory. Analytical chemistry 83:67-76.

Arnott WP, Hamasha K, Moosmuller H, Sheridan PJ, Ogren JA. 2005. Towards aerosol light-absorption measurements with a 7-wavelength aethalometer: Evaluation with a photoacoustic instrument and 3-wavelength nephelometer. Aerosol Sci Tech 39:17-29.

Chow JC, Watson JG, Chen LWA, Chang MCO, Robinson NF, Trimble D, et al. 2007. The improve-a temperature protocol for thermal/optical carbon analysis: Maintaining consistency with a long-term database. J Air Waste Manage 57:1014-1023.

Gautier L, Cope L, Bolstad BM, Irizarry RA. 2004. Affy - analysis of affymetrix genechip data at the probe level. Bioinformatics 20:307-315.

Gentleman RC, Carey VJ, Bates DM, Bolstad B, Dettling M, Dudoit S, et al. 2004. Bioconductor: Open software development for computational biology and bioinformatics. Genome Biol 5.

Grabowsky J, Streibel T, Sklorz M, Chow JC, Watson JG, Mamakos A, et al. 2011. Hyphenation of a carbon analyzer to photo-ionization mass spectrometry to unravel the organic composition of particulate matter on a molecular level. Anal Bioanal Chem 401:3153-3164.

Keskinen J, Pietarinen K, Lehtimaki M. 1992. Electrical low-pressure impactor. J Aerosol Sci 23:353-360.

Kooter IM, Alblas MJ, Jedynska AD, Steenhof M, Houtzager MM, Ras M. 2013. Alveolar epithelial cells (a549) exposed at the air-liquid interface to diesel exhaust: First study in tno's powertrain test center. Toxicology in vitro : an international journal published in association with BIBRA 27:2342-2349.

Lyyranen J, Jokiniemi J, Kauppinen EI, Backman U, Vesala H. 2004. Comparison of different dilution methods for measuring diesel particle emissions. Aerosol Sci Tech 38:12-23.

Mülhopt S, Krebs T, Paur H-R. 2008. Online dose determination for in vitro experiments with nano particles in the karlsruhe exposure system. Toxicology Letters 180, Supplement:S224.

Ong SE, Mann M. 2006. A practical recipe for stable isotope labeling by amino acids in cell culture (silac). Nature protocols 1:2650-2660.

Orasche J, Schnelle-Kreis J, Abbaszade G, Zimmermann R. 2011. Technical note: In-situ derivatization thermal desorption gc-tofms for direct analysis of particle-bound non-polar and polar organic species. Atmos Chem Phys 11:8977-8993.

Prytherch Z, Job C, Marshall H, Oreffo V, Foster M, BeruBe K. 2011. Tissue-specific stem cell differentiation in an in vitro airway model. Macromolecular bioscience 11:1467-1477.

R-Core-Team. 2012. R: A language and environment for statistical computing, r foundation for statistical computing. Vienna, Austria.

Reischl GP. 1991. Measurement of ambient aerosols by the differential mobility analyzer method - concepts and realization criteria for the size range between 2-nm and 500-nm. Aerosol Sci Tech 14:5-24.

Schauer C, Niessner R, Poschl U. 2004. Analysis of nitrated polycyclic aromatic hydrocarbons by liquid chromatography with fluorescence and mass spectrometry detection: Air particulate matter, soot, and reaction product studies. Anal Bioanal Chem 378:725-736.

Welthagen W, Schnelle-Kreis J, Zimmermann R. 2003. Search criteria and rules for comprehensive two-dimensional gas chromatography-time-of-flight mass spectrometry analysis of airborne particulate matter. J Chromatogr A 1019:233-249.

Wiedensohler A, D. Orsini, D. S. Covert, D. Coffmann, W. Cantrell, M. Havlicek, F. J. Brechtel, L. M. Russell, R. J. Weber, J. Gras, J. G. Hudson and M. Litchy 1997. Intercomparison study of the size-dependent counting efficiency of 26 condensation particle counters. Aerosol Sci Technol 27:19.

Wisniewski JR, Zougman A, Mann M. 2009. Combination of fasp and stagetip-based fractionation allows in-depth analysis of the hippocampal membrane proteome. Journal of proteome research 8:5674-5678.
